# Comparison of national level biomass maps for conterminous US: understanding pattern and causes of differences

**DOI:** 10.1186/s13021-016-0060-y

**Published:** 2016-08-26

**Authors:** N. Neeti, R. Kennedy

**Affiliations:** 1 Department of Natural Resources, TERI University, New Delhi, India; 2 College of Earth, Ocean, Atmospheric Sciences, Oregon State University, Corvallis, OR USA

**Keywords:** CMS, AGB, FIA, Carbon

## Abstract

**Background:**

As Earth observation satellite data proliferate, so too do maps derived from them. Even when two co-located maps are produced with low overall error, the spatial distribution of error may not be the same. Increasingly, methods will be needed to understand differences among purportedly similar products. For this study, we have used the four aboveground biomass (AGB) maps for conterminous US generated under NASA’s Carbon Monitoring System. We have developed systematic approach to (1) assess both the absolute accuracy of individual maps and assess the spatial patterns of agreement among maps, and (2) investigate potential causes of the spatial structure of agreement among maps to gain insight into reliability of methodological choices in map making.

**Results:**

The comparison of the four biomass maps with FIA based total biomass estimates at national scale suggest that all the maps have higher biomass estimate compared to FIA. When the four maps were compared among each other, the result shows that the maps S and K have more similar spatial structure whereas the maps K and W have more similar absolute values. Although the maps K and W were generated using completely different methodological workflow, they agree remarkably. All the maps did well in the dominant forest type with maximum agreement between them. The comparison of difference between maps S and K with regional maps suggests that these maps were able to capture the disturbance and not so much regrowth pattern.

**Conclusions:**

The study provides a comprehensive systematic approach to compare and evaluate different real data products using examples of four AGB maps. Although ostensibly the four maps map the same variable, they have different spatial distribution at different scale. Except the 2003 map, one can use other maps at the coarser spatial resolution. Finally, the disparate information available through different maps indicates a need for a temporal framework for consistent monitoring of carbon stock at national scale.

## Background

Because forests provide important ecosystem services and play a role in the global carbon cycle, forest characteristics such as biomass, tree height, and percent forest cover have been mapped using a variety of remote sensing techniques [1–7]. As data increase in availability, and as mapping techniques proliferate, many similar products are becoming available for the same region—often at different spatial scales and derived using different techniques. In an ideal world, multiple mapped estimates of the same biophysical variable should largely agree in the same location and time. Presumably, all credible maps are verified against a more reliable source to estimate overall error, but the spatial distribution of errors may not be the same for different maps. Thus, when two maps of the same quantity are compared at a given location, they may disagree. From an end-user perspective, this is a problem: different maps may have quite different implications for carbon accounting, for example, but users are given no guidance about which map to choose. From a scientific perspective, this may be an opportunity: patterns of disagreement among maps may provide insight into how datasets and techniques perform under varying conditions.

Reconciling maps will become an increasingly important activity within NASA’s Carbon Monitoring System (NASA-CMS) [8]. NASA-CMS is a broad initiative to apply NASA’s synoptic view of Earth systems to the monitoring of carbon. NASA-CMS activities run the gamut from local-scale mapping of carbon state [9, 10] to global scale mapping of carbon flux [11]. Often, projects at different scales produce similar products, or products that could be compared against other projects through simple manipulation (e.g., multiple estimates of biomass over time could be compared against estimates of flux). As a mix of both regional scale and global scale projects, NASA-CMS will be increasingly faced with disparate estimates of carbon states and fluxes at different scales.

Recognizing that different estimates of carbon-related maps co-occur, several researchers have recently reported on comparisons among different biomass maps [12–14]. In some cases, comparisons among maps were made with fine-resolution reference data (e.g., [13, 14]), but such data are not always available. In other cases, maps were compared at the same spatial resolution (e.g., [12]) but this precludes comparisons generated at different resolution. In fact, there are often situations where we need to identify a map which is most close to the truth in the absence of fine-resolution reference data while understanding various methodological and input grain size differences between various available maps. Indeed, the notion of multiple maps may imply that one is better, or that one more closely matches truth, but such a uniform truth is rarely feasible, and therefore we need spatial distribution of uncertainty. In practice, the assessment of spatially-distributed map data can be considered an effort to paint a picture of spatial uncertainty which is carried out by describing relative error among maps or measurements using a suite of complementary quantitative measures [15]. However, there exist several challenges in assessing a map and comparing different maps. First, the unit of analysis in a raster-based map is a square pixel that is arbitrary when we compare to any phenomenon in the landscape. Second, raster based maps are often generated at different scales, so comparison requires cell by cell alignment while ensuring the information in individual maps remain same. Moreover, most of the techniques developed to compare real variable maps can be used only for pairwise comparison, limiting the ability of simultaneous comparison of multiple maps to assess the spatial pattern of similarity and dissimilarity.

As the NASA-CMS matures, mapped estimates of both carbon biomass and flux will be available at different scales for the same location, requiring quantitative comparisons among many maps. Indeed, NASA-CMS has already produced one forest biomass map for the conterminous United States, but already several versions existed developed by other groups. For users, guidance must be given about the relative merits of these maps under different conditions, and under what conditions a given map may be avoided. For developers and scientists, however, comparisons should be structured to leverage the differences in methods to provide insight into possible improvements or best practices. Thus, using these several forest biomass maps as a test case, we report on strategies and methods both to describe differences among maps for users, and to evaluate possible sources of disagreement.

We therefore had two broad objectives:

1. Descriptive: Assess both the absolute accuracy of individual maps and assess the spatial patterns of agreement among maps.
2. Evaluative: Investigate potential causes of the spatial structure of agreement among maps to gain insight into reliability of methodological choices in map making.

To achieve these objectives, we introduce a comprehensive and systematic approach to compare multiple maps generated at different scales (extent and spatial resolution). This comprehensive approach not only evaluates accuracy of individual maps while describing the similarity and dissimilarity between the spatial structure of various maps, but also systematically explores effects of scale and various causes of change.

## Methods

**Fig. 1:**
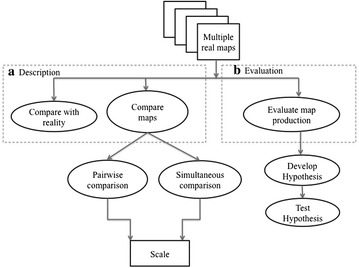
Overall systematic approach in reconciling maps. **a** Descriptive phase involves assessing accuracy of individual maps and both pairwise and simultaneous comparison of multiple maps, **b** evaluation phase involves detailed assessment of statistical approach to understand implications of identified sources of differences

Our systematic approach consists of several steps (Fig. 1). The foundational step is a thorough assessment of key map-making steps. This provides a context for the descriptive phase, and helps guide hypotheses to be tested in the evaluative phase.

Next, the descriptive phase seeks to quantify how well individual maps agree with reference data and with each other. Two key strategies are important. First, in addition to evaluating pairwise differences, as is done in other comparative studies, we advocate simultaneous comparison across all maps to understand underlying spatial patterns of agreement and potentially isolate outlier maps. Second, we aggregate our measures of agreement and disagreement to ecologically coherent mapping regions, recognizing that some maps may perform better in particular ecological contexts and that users may only be interested in this regional scale.

Finally, the evaluative phase seeks to test specific sources of error in the map making process. Here, we must draw inference from comparisons without the benefit of manipulation. Thus, rather than simply developing a range of comparative metrics, this phase places comparisons into specific test of methodological contrast among maps. The key is to hypothesize how differences in methodology would lead to specific differences among pairs of maps, and then test those hypotheses. It is the combination of metric and map combination that allows greater inference: the observation of an effect in a single map is likely uninformative, but hypothesis-driven comparison of that effect between maps may provide greater insight.

### Assessment of map-making steps

**Fig. 2:**
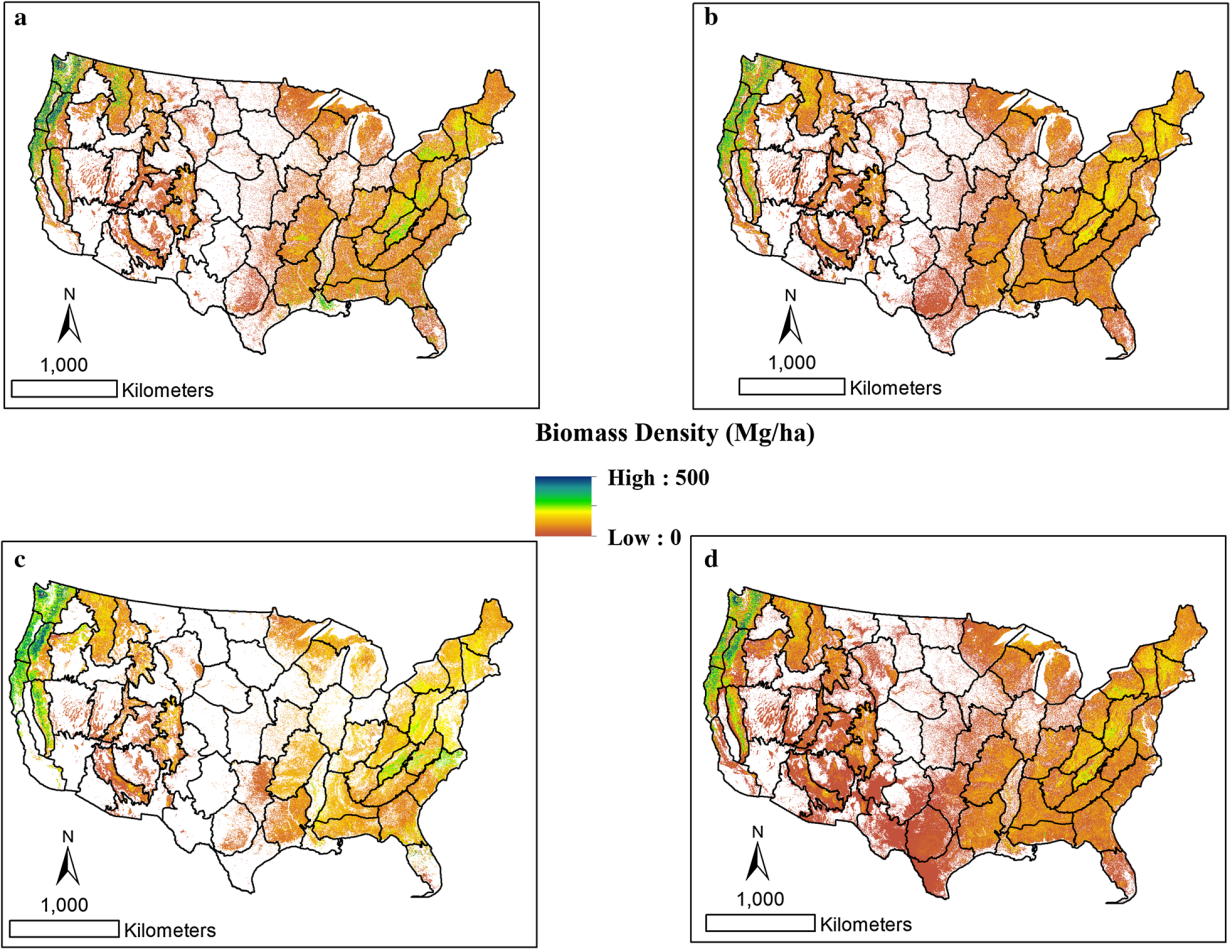
Aboveground biomass (AGB) density maps for conterminous US **a** Saatchi et al. [16] ‘S’, **b** Kellndorfer et al. [17] ‘K’, **c** Blackard et al. [18] ‘B’, **d** Wilson et al. [19] ‘W’

We examined the four national-scale maps of forest biomass that were available at the beginning of our study (Fig. 2). Papers detailing map production are given by Saatchi et al. [16], Kellndorfer et al. [17], Blackard et al. [18], and Wilson et al. [19]. For parsimony, we hereafter refer to these maps by their first letter, i.e. S, K, B, and W, respectively. All maps are generally considered usable, either through self-reported accuracies or through community use. Map W is reported to have strong agreement with plot based estimates of biomass (agreement coefficient ~0.99) and to have a strong goodness of fit (within 90 % confidence interval). The average absolute error for the map B ranges between 40 and 60 metric tons per hectare, except for the higher-biomass areas of the Pacific Northwest (163 metric tons per hectare). The accuracies for map S and K are not known, but they, like all of the maps, are already being used as input in different studies [10, 20, 21].

Like many spatial mapping exercises in remote sensing, the general approach for producing AGB maps is to extrapolate high quality training data from a small sample of locations to a large, contiguous space. Typically, values measured at the training samples are linked with data values from geospatial datasets at the locations where they intersect. Statistical models built at those locations are then used to extrapolate to the rest of the map. Maps can differ in how they handle training data, which geospatial datasets are used, and what statistical models are built.

We identified six methodological sources of disagreement in how the four biomass maps were generated (Table 1). For each, we developed expectations for how that difference may manifest itself in the resultant maps. The first source is the forest mask used to define the total area for which biomass is estimated. Conservative forest masks would lead to lower estimates of total biomass, but a more liberal forest mask would include a higher proportion of marginal forest and thus likely reduce average biomass density. The second source of potential disagreement between the maps is spatial resolution. Map resolution interacts with the fundamental spatial structure of the landscape: If the former is finer than the latter, the map can adequately capture the range of variability of the landscape. A third contrast among maps is the remote sensing source data used for extrapolation. Maps using optical data may be less able to capture high biomass than maps using radar data, as optical data are known to saturate at lower biomass compared to radar (longer wavelength such as L- and P-band) [22–25]. The sensitivity of L- band SAR data for biomass estimation increases if used along with forest height generated using InSAR [25]. The fourth source of difference is the statistical technique used for extrapolation (parametric vs. non-parametric). Non-parametric techniques could be expected to perform better at extreme values (low and higher biomass region) than parametric techniques, especially if the extreme values deviate greatly from a normal distribution. The fifth source of difference is year of mapping. Maps produced in different years would be expected to differ both because of intervening disturbance and growth, and because the pool of training data could be different.

**Table 1:** Sources of disagreement among the four national AGB maps

AGB maps	Forest mask	Grain size (m)	Spatial predictors	Extrapolation technique (parametric/non-parametric)	Year of generation of map	Allometric equation
Saatchi et al. [16] (S)	NLCD 2006	93	F(MODIS, Landsat, L-band PALSAR, GLAS, topography)	Maximum Entropy (Parametric)	2005	Component ratio method
Kellndorfer et al. [17] (K)	NLCD 2001	30	F(C-band InSAR, Landsat, NLCD canopy density, structure, SRTM)	Regression tree (Random forest, Non-parametric)	2000	Regional method
Blackard et al. [18] (B)	In house	250	F(MODIS, climate variables variables, topography)	Regression tree (Cubist, Non-parametric)	2003	Regional method
Wilson et al. [19] (W)	No Forest mask	250	F(MODIS, climate variables, topography, level III ecoregions)	Phenological gradient nearest neighborhood (Semi-parametric/Semi-nonparametric)	2009	Component ratio method

The sixth area of potential disagreement rests not with the methodologies to extrapolate, but with the training data themselves. In the case of forest AGB in the conterminous US, the training data come from the US Forest Service’s Forest Inventory and Analysis (FIA) program. At each of thousands of field plots, field crews measure details of trees using a regular sampling and mensuration protocol. These raw tree data can then be converted to estimates of plot-level biomass using allometric equations that relate tree characteristics to known biomass measured destructively at a sample of locations (and often as an entirely separate effort). Allometric equations varied across the four maps studied here, with some using regional-specific equations and others a more nationally-consistent component ratio method (CRM) [26]. The CRM approach has been shown to lead to lower biomass estimates then the regional approach [26], and thus maps based on the CRM would thus be expected to show an overall bias toward lower values.

### Descriptive comparisons

Descriptive comparisons provide users guidance about which maps are more accurate, and where on the landscape (at both national and regional scales) the maps agree and disagree when analyses carried out at pixel level.

#### Comparison with ground reference data

Because the FIA program is tasked with providing defensible estimates of forest resources at the national scale, estimates from FIA plot data are the de facto standard against which any forest resource maps must be compared. Although FIA plot data are used as inputs in various points of the mapping process for all four national maps tested here, this does not guarantee that summarized estimates of biomass will agree, since maps extrapolate the FIA information differently.

Comparison with FIA data requires appropriate use of those data. The goal of the FIA sampling design is to provide good estimates at aggregated administrative levels: The smallest unit is typically the US county or parish, but state-level comparisons are more common and robust, especially when the analyses is carried out at the MODIS scale [27]. Additionally, normal users do not have access to actual plot locations, and thus data are typically available and readily usable only at county or larger scales.

Thus, for our comparisons we compared plot and map data at the aggregation level of the state. Map data aggregation was a simple matter of summing biomass density estimates to the state level, utilizing each map’s own forest mask for the aggregation footprint. For plot data, we acquired state level FIA AGB data from the FIA 2013 database [28] at the appropriate time step for the FIA collection strategy of the state. For forests in the eastern US, we used the time frame of 2003–2007, as that was the most consistent time frame without double counting any trees across different states. For forests in the western US, which are on a 10 year repeat cycle, we used data from 2000 onwards for the west side as that was the most consistent data available through the website across all the states at the time of analyses. The estimates have already converted the plot-level tree measurements to the plot scale, and then used sample-design considerations to scale the plot-level data to the state level. Three considerations of the FIA data are relevant for later comparisons. First, the FIA uses the CRM approach to calculate biomass from tree measurements. Second, the inventory cycles occur at 5 and 10 year cadences in the east and west, respectively, meaning that an estimate at a given time will be a different mix of older and more recent plots in the east and the west. Finally, the extrapolation scaling factors are based on forestland masks developed by the US Forest Service (and also used in the B and W maps).

Once biomass had been aggregated, we produced three descriptive products. First, we summed forest biomass among all maps and all plots at the conterminous US scale. Second, we used simple regression of aggregated map biomass at the state level against FIA plot biomass (n = 48 states in conterminous US). Finally, we calculated the Wilmott’s index of agreement (d):

$$d = 1 - \frac{{\mathop \sum \nolimits_{i = 1}^{n} (X_{i} - Y_{i} )^{2} }}{{(|X_{i} - \underline{X} | + |Y_{i} - \underline{X} |)^{2} }}X$$

where X_i_ was the biomass estimate for state *i* estimated from FIA plot data and Y_i_ was the same estimated from the maps [29]. The X variable is considered to be the truth variable, which is appropriate here because FIA plot data are the national standard for forest monitoring. Wilmott’s index of agreement is symmetric, bounded and does not over penalize for disagreement. High agreement is indicated by values close to 1.0. There exist many other indices for such pair wise comparison such as mean square error (MSE) and its root (RMSE) to more specialized metrics [e.g., agreement coefficient (AC)] to comparing distribution. The indices such as MSE, RMSE have been critiqued for being unbounded and asymmetric [30] while the AC is found to highly sensitive to outlier. The comparison of distributions could simply yield the uninteresting finding of differences with little insight on the reason behind the differences. Spatial structure of agreement and disagreement is critically useful for understanding whether disagreement is potentially related to specific issues.

#### Comparison of multiple maps

After descriptive comparison against a trusted data source (FIA based estimates), the next step was to show where maps agreed and disagreed. We used principal components analysis (PCA) for simultaneous comparison among all maps. PCA is a spectral decomposition technique commonly used in remote sensing to remove the redundancy from multi-spectral images, but it can also be used to identify the dominant pattern common among various maps [31]. The six orientation modes (O, P, Q, R, S, T) commonly used for PCA differ in their definition of statistical variables and observations [32]. In this case, statistical observations are samples in space and statistical variables are various continuous maps, therefore, R-mode PCA is used for the analysis.

Implementation of the PCA took place in the R statistical package [33], and required some basic preparation of the datasets. First, because the maps reported biomass in different unit systems and different map projections, we aligned all biomass map values to the same system at the pixel scale, and then aggregated (taking mean of x by x window size followed by nearest neighborhood resampling, x = 8 for map S, x = 2) the finer-scale maps (S and K) to the 240 m grain size of the coarser maps to make them comparable to the other two maps. The coarser resolution maps (B and W) were resampled from 250 m to 240 m resolution by using nearest neighborhood approach. Second, we clipped all maps to the forest mask area common to all forest masks, as “no-data” would not be informative in the PCA analysis. Finally, we stacked all four maps into a single, four-layer image. Once these preparatory steps had been taken, we ran the PCA analysis on both the entire conterminous US data (at the 240 m pixel scale) and at the scale of each of the 66 mapping regions [34]. The regional scale analysis is useful because the PCA statistical space is defined by the range of variation in the whole dataset, and thus the broad US-wide comparison may obscure patterns that would be relevant to users at the regional scale.

Results of the PCA were interpreted in two ways. First, spatial patterns in the first and second axis images show where on the landscape the maps agree and disagree. Because PCA axis 1 identifies the vector through the multivariate space that explains the most variation, it can be interpreted as the overall pattern of biomass agreement across all maps. PCA axis 2 is orthogonal to the first axis, and thus indicates the dominant sources of disagreement among the maps. Second, the correlation coefficients of each map contributing to those two axes can give insight into which maps are agreeing or disagreeing with the other maps. A map with high correlation coefficient on the first axis is one whose spatial patterns agree with the other maps; a map with high correlation coefficient on the second axis is one whose spatial patterns disagree with the other maps.

#### Scale of agreement

If maps generally agree with truth data when aggregated to the mapping region scale, but show patterns of disagreement at the pixel scale, then a natural question from users is whether intermediate scales of resolution may make maps more comparable. To evaluate this question, we conducted pair-wise map comparison to analyze the impact of spatial resolution on map agreement. As with the PCA analysis (‘‘comparison of multiple maps’’ section), we first ensured that all maps were clipped to the same footprint and were at the same starting pixel resolution (acknowledging that the 30 m and 90 m products were already degraded for the first comparison), and then we aggregated from the starting 240 m resolution to pixel resolutions of 480, 720 and 960 m. For each of those resolutions, we evaluated paired map agreement with the Wilmott’s index of agreement at mapping region scale, as all the pixels in a mapping region are expected to have similar ecological condition. Critically, because no map could be considered the truth map, we conducted this analysis on all pairwise comparisons. High median d-scores across all mapping regions suggest consistent strong agreement, while high range of d-scores suggests spatial patterns of variability across mapping regions.

### Evaluative comparisons

Evaluative comparisons were designed to test whether patterns of agreement and disagreement could provide insight into sources of error in map production. Because each map was produced using a different suite of methods (Table 1), each map needed to be compared individually to the other maps. To test the six different possible sources of differences, we used a range of different pairwise map comparisons, both on the original data and on maps of differences between pairs of maps. Additionally, for certain tests we brought in ancillary data on forest type, topographic position, and regional biomass time series. We first describe the basic data manipulations, and then describe how these were used to evaluate the six different sources of map difference.

#### Data manipulations

##### Cumulative biomass

The simplest data manipulation was simple summing of biomass across all pixels in a given map to estimate total forest biomass at the national scale. Total biomass at the national scale had already been calculated for the descriptive phase (see section on ‘‘cumulative biomass’’ section).

##### Mapping region scale biomass density

Within each mapping region, we calculated the cumulative frequency distribution of biomass density values of a map for all pixels in each map’s forest mask. From these, we identified the 10th-, 50th- and 90th percentile values for the mapping region. We also calculated mean biomass density by mapping region.

##### Difference maps

For all pairwise combinations of the four national-scale maps, we developed maps of biomass difference at the pixel scale. As with the PCA analysis, the maps were first clipped to a common forest mask, and if the pixel sizes of the two maps were different, the higher resolution map was resampled and aggregated to the lower resolution map cell size (see details on aggregation above). Differencing was achieved using simple image algebra on a cell by cell basis. Spatial patterns in the difference maps were then related to spatial patterns of ancillary data.

##### Ancillary data

We sought to understand whether spatial patterns in map differences were related to spatial patterns in other geospatial datasets. For these, we obtained the spatial predictor data layer and manipulated the map as necessary (using mean/mode aggregation and/or nearest neighborhood resampling) to ensure cell alignment among maps. We obtained through the ORNL website [35] a forest type land cover map with cells of 250 m resolution, for the nominal mapping year 2001. We utilized all forest cover types for analysis. Separately, we obtained a forest age map with cell size of 1 km for the nominal mapping year 2006 [36] and grouped pixels into young (<40 years old), immature (40–80 years old), mature (80–140 years), and old (>140 years). Finally, for topographic variables, we obtained SRTM elevation at 30 m resolution and derived slope. We grouped pixels according to three elevation categories (<500 m, 500–1500 m, and >1500 m), and, where appropriate, two slope categories: relatively benign (0 to <30 degree slope) and extreme (30–50 degree slope). Within mapping regions of interest, we summarized the difference maps according to the zones delineated by the groups in those ancillary data sources.

To assess impact of year of map generation, we required ancillary biomass map data where technique of production was held constant across time. At present no such map exists at the national scale, and thus we used a regional-scale map product [37]. That product generated yearly biomass estimates from 1990 to 2010 for the high-biomass states of Washington, Oregon, and California. Because the core of that approach was a change detection approach at the Landsat scale, those maps explicitly represent the effects of growth and disturbance on biomass. We refer to these maps as KennXXXX, where XXXX represented the year of map. We differenced KennXXXX maps for the years corresponding to each of the map pairs in the national efforts, and compared distributions of the differences in the consistently-produced maps to the differences in distributions in the national maps. Note that we not using this map as a truth dataset, but simply as a means of holding constant the means of production across time to isolate the impacts of growth and disturbance.

#### Evaluative tests

The data manipulations were then used in evaluative tests of each of the six identified sources of map error. We emphasize that evaluative tests utilize metrics that by themselves could be uninformative—they key is to compare the metrics among map pairs, driven by hypotheses about how map production could lead to differences.

##### Forest mask

Each map used a slightly different mask to identify potential forested pixels. If the forest mask were the only factor affecting map results, we would expect that maps with a conservative forest mask would show a lower total biomass when aggregated to the national scale.

To test, we simply summed biomass nationally for all four maps and evaluated relative to the forest mask area of each map.

##### Spatial resolution

Spatial resolution determines the scale of pattern which can be captured by a given map. If spatial patterns of forest biomass vary meaningfully at a scale finer than that of a given map, that map will be unable to capture the high and low biomass values, and thus would be expected to have a compressed range of biomass relative to the actual landscape [24]. When comparing among maps of different resolution, the impact of resolution will matter if the scale of meaningful variation is intermediate between resolutions of the maps.

To test, we compared 10th and 90th percentile values by mapping region (‘‘mapping region scale biomass density’’ section) for all pairs of maps. If pixel resolution were a driving factor, we would expect to see compressed ranges in both the W and B maps relative to the S and K maps.

##### Source sensor data

Because the signal retrieved from a passive optical sensor typically saturates at biomass levels lower than that of an active sensor such as radar (e.g., L-Band SAR, C-band InSAR) [26, 27], we might expect that maps involving radar would be able to track biomass better in high-biomass mapping regions even though there may be difference between various radar data depending on frequency and polarization. To test, we compared the 90th percentile values by mapping region for all pairs of maps. If sensor source were a driving factor, we would expect the W and B maps to show a compressed upper end relative to the S and K maps.

Similarly, because older forests have more structurally complex canopies, we might expect an active-sensor approach to better capture biomass in older forests, or in generally higher-biomass forest types [38]. To test, we compared difference maps grouped by forest type and age for mapping regions where high biomass and older forests were more prevalent. If active sensors performed better in these systems, we might expect the S and K maps to show higher biomass (net positive difference) vs the B and W maps in older forests and in higher biomass type forests.

Finally, because of the side-looking nature of radar, we might expect the radar-derived maps to perform more poorly in topographically complex areas [39]. To test, we compared difference maps grouped by elevation and slope categories (noted in section on the ancillary data in the method above). If error introduced by side-looking radar were an issue, we would expect the S and K maps to show greater difference with the optical maps in either high elevation or high slope areas.

##### Extrapolation technique

When linking the observed covariate (here, biomass) to predictor data (here, geospatial data), parametric regression techniques explicitly attempt to minimize variability in the covariate, often resulting compressed prediction ranges when extrapolation is later performed [40] low values are overestimated and high values are underestimated. Non-parametric approaches may not suffer from this extrapolation issue. To test, we examined the 10th and 90th percentiles of biomass distributions. If extrapolation technique were an issue, we would expect the W and S maps to show ranges compressed relative to the K and B maps.

##### Nominal year of map

National maps were produced in different years, and thus some differences between any pair of maps would be caused by intervening growth and disturbance in the period between the two maps. Thus, the observed difference between maps convolves both map production differences and real differences in the landscape. To test, we compared difference maps to differences in the KennXXXX map pairs for the same period. Because the KennXXXX maps explicitly capture disturbance and growth processes using a consistent technique, they serve as a proxy for the expected differences between maps for any given pair of years. If two maps agree but differ only in disturbance and growth, we would expect the distributions of biomass difference to be similar to that in the proxy maps. Substantial departures from that expected distribution would indicate that year of map production was not the only cause of difference in the maps.

##### Allometric equations

Because the CRM method used nationally has been shown to produce lower estimates of biomass than regionally-specific allometric equations, we would expect mean and median values of biomass to be lower in those maps using CRM approaches. To test, we compared median and mean values by mapping region. If allometric equations were a critical difference, we would expect the S and W maps to be consistently lower than the K and B maps.

## Results and discussion

### Descriptive comparisons

#### Comparison with ground reference data

All four maps estimate slightly higher national AGB than does the summed FIA plot data based estimates for conterminous US (Table 2). Of the four, map S’s estimate is most similar to FIA and map B’s most dissimilar.

**Table 2:** Total forest area and biomass for conterminous US for the four AGB maps based estimates and FIA based estimates

AGB map	Forest area (Million Ha)	Total biomass (Gt)
K	414.8	27.4
B	260.47	29.6
S	350.23	26.7
W	486.14	27.2
FIA	–	25.4

**Fig. 3:**
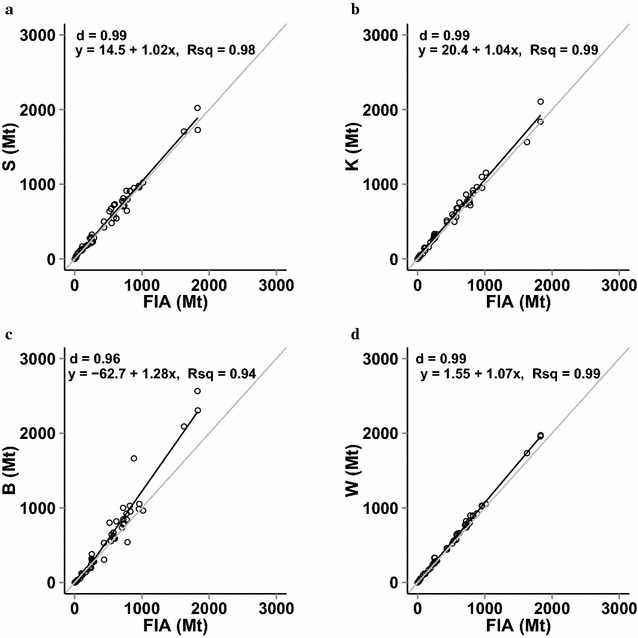
Comparison between FIA plots based estimates and the four AGB maps at state scale: **a** FIA vs S, **b** FIA vs K, **c** FIA vs B, **d** FIA vs W

When regressing biomass on a state-by-state basis (Fig. 3), map S shows the closest alignment with the 1:1 line (slope: 1.02), but with greater scatter than both maps K and W (r^2^ = 0.98, 0.99). Map B remains substantially different at the state scale, particularly for medium to high biomass states. Wilmott’s *d* suggests that map W is most similar to the FIA estimates at state scale, followed closely by map S.

#### Comparison across multiple maps

**Fig. 4:**
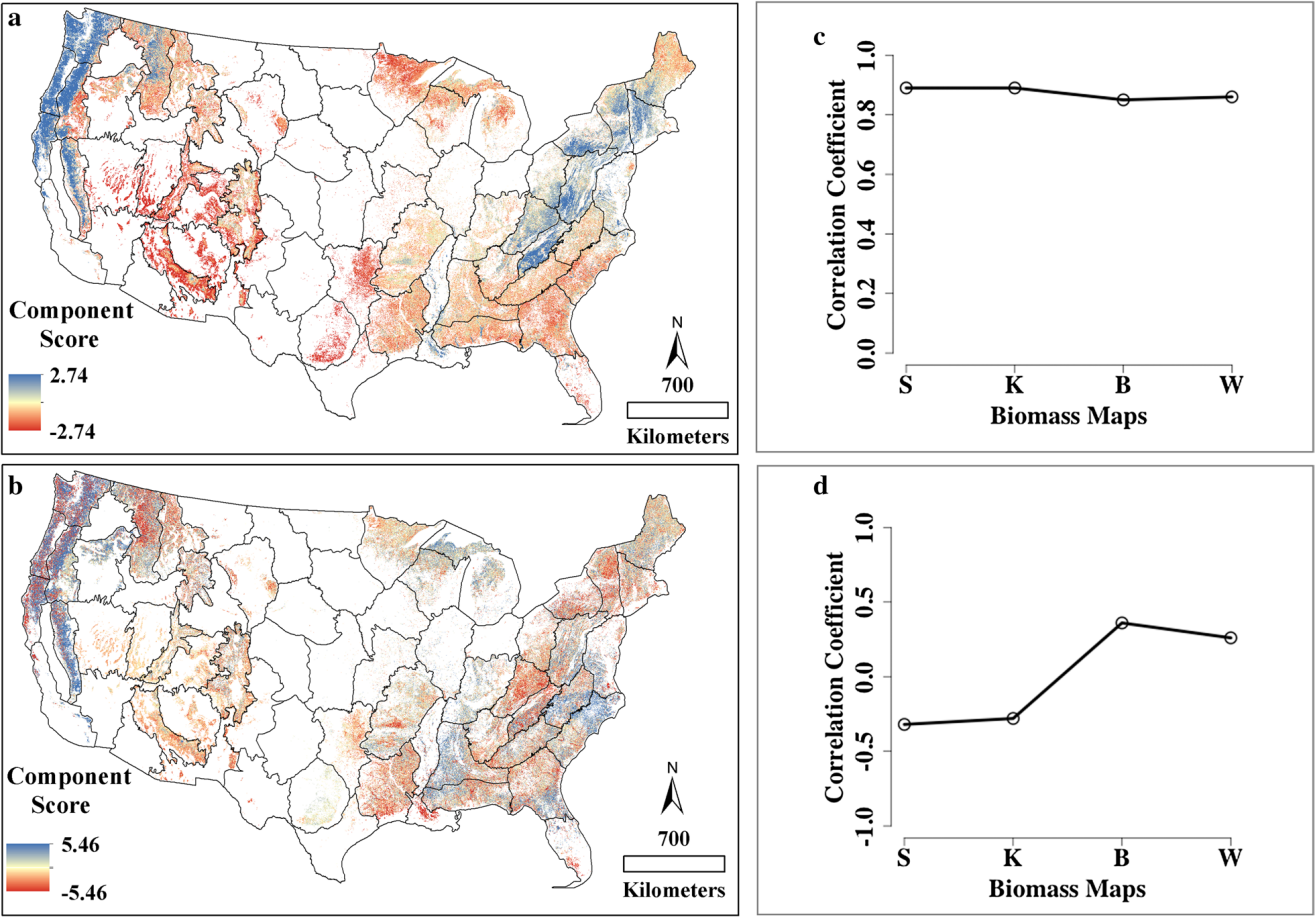
Spatial pattern of agreement and disagreement among four AGB maps at pixel scale for conterminous US generated using PCA **a** Principal Component 1 (PC1) score. **b** Principal Component 2 (PC2) score. **c** Correlation between PC1 score and the four AGB maps. **d** Correlation between PC2 score and the four AGB maps

**Fig. 5:**
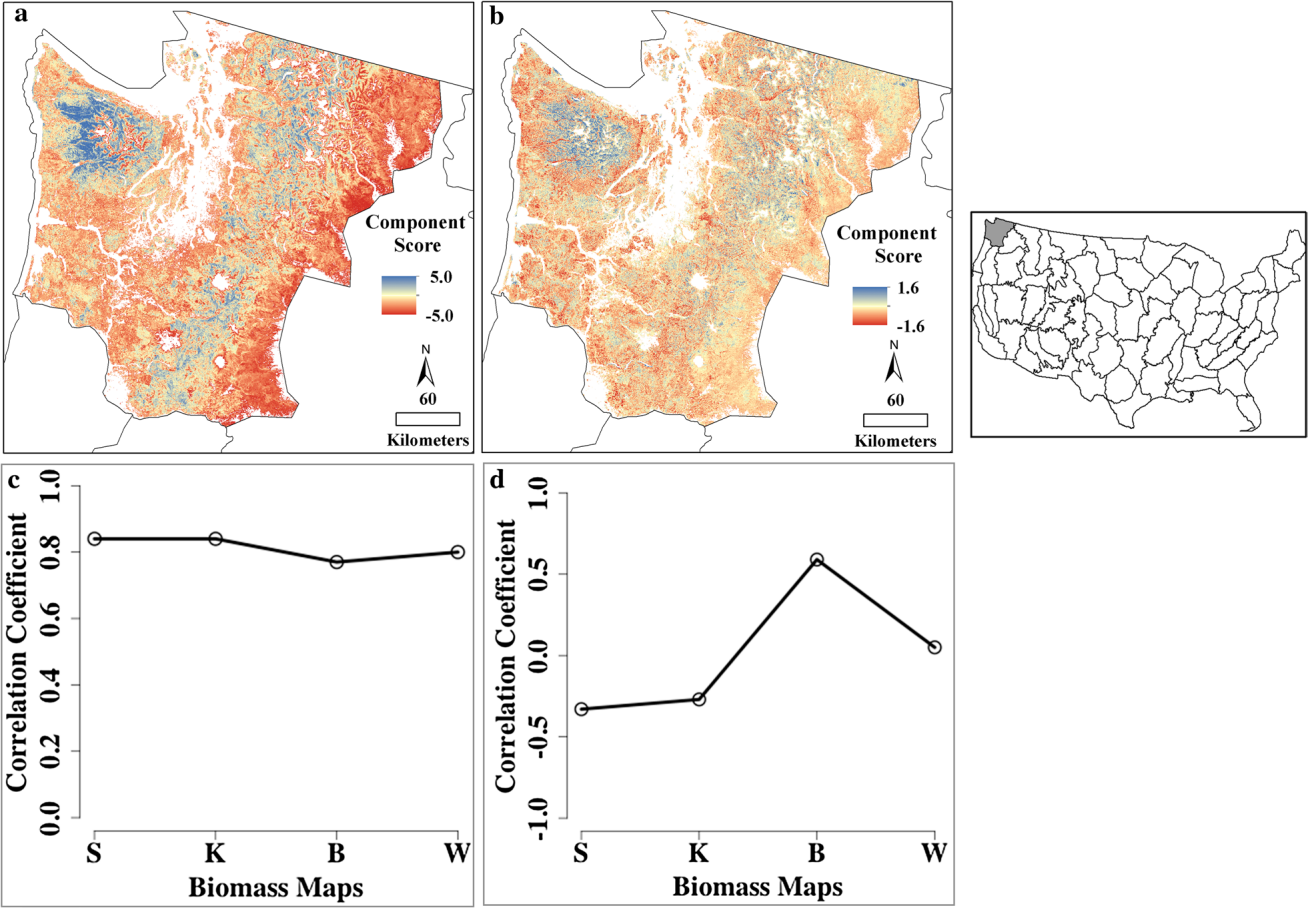
Spatial pattern of agreement and disagreement among four AGB maps at pixel scale in mapping zone 1 generated using PCA. **a** Principal Component 1 (PC1) score. **b** Principal Component 2 (PC2) score. **c** Correlation between PC1 score and the four AGB maps. **d** Correlation between PC2 score and the four AGB maps

**Fig. 6:**
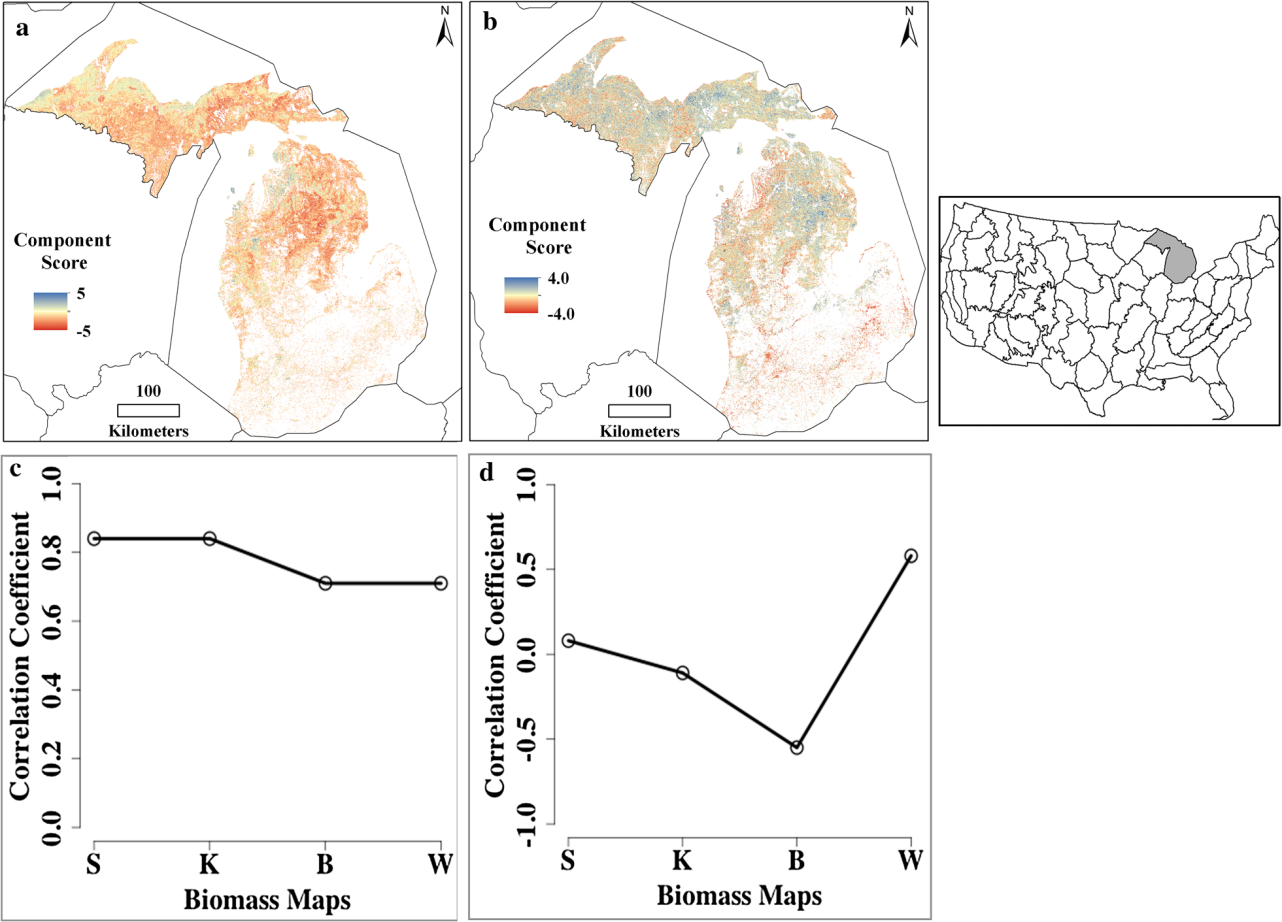
Spatial pattern of agreement and disagreement among four AGB maps at pixel scale for mapping zone 52 generated using PCA. **a** Principal Component 1 (PC1) score. **b** Principal Component 2 (PC2) score. **c** Correlation between PC1 score and the four AGB maps. **d** Correlation between PC2 score and the four AGB maps

The national-scale PC1 image shows patterns of biomass that are generally in agreement among all four maps (Fig. 4a), with correlations strong between PC1 and all four source maps (Fig. 4c). As expected, forest biomass is highest in west-coast forests, lower in parts of the mountainous eastern interior forests and lowest in the interior west and north central states. Patterns of biomass disagreement (PC 2) are more spatially heterogeneous (Fig. 4b), and appear to be dominated by map B (Fig. 4d). The difference of map B is in the PCA analysis is consistent with its difference against the other maps in the state level comparison with FIA plot data (Fig. 3). Relative to PC comparisons at the national scale, mapping-region scale PC analyses can show more nuanced or even opposing patterns. Mapping region 1 (Washington state) and mapping region 52 (Wisconsin and Michigan state) provide useful examples. In mapping region 1 (Fig. 5), patterns of biomass agreement (PC 1, Fig. 5a, c) are as expected, with high biomass forests in the wetter western portions of the state and lower in the drier eastern portions of the state. Consistent with the national scale analysis, Map B dominates the disagreement vector (PC 2, Fig. 5b, d). However, in mapping region 52, Map W and map B dominate the disagreement vector (PC 2, Fig. 6b) even though the patterns of biomass agreement are largely as expected (Fig. 6a, c). The map W and map B has completely opposing patterns in the mapping region, and the spatial pattern is not at all related to the other two maps (Fig. 6b, d). Thus, the guidance for which map is less reliable may vary with mapping region, and points to the need for local users to develop local-scale comparisons.

#### Scale of agreement

**Fig. 7:**
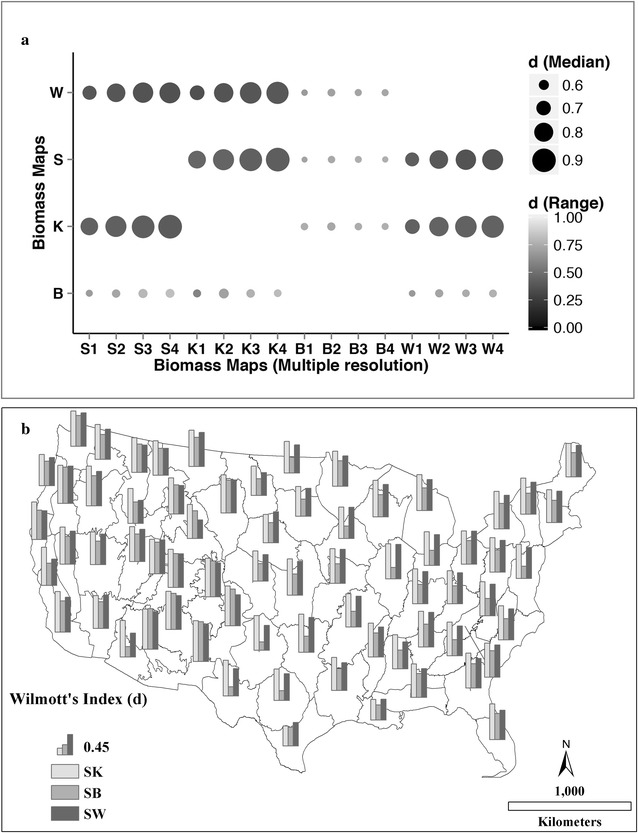
Pairwise comparison between the four AGB maps **a** multiple resolution comparison at mapping zone scale where X1 = 240 m, X2 = 480, X3 = 720 m, X4 = 960 m; X = AGB map, **b** Comparison at 240 m at pixel level within each mapping zone

For all maps but Map B, the Wilmott’s measure of agreement between pair-wise maps suggests that agreement increases at coarser spatial resolution, potentially leveling off at the top end of the range of spatial resolution tested here (Fig. 7a). There is high consistency across all pair-wise comparisons at multiple spatial resolution (range of d values less than 0.4) except for the comparison with the map B. For Map B, similarity did not change with increasing resolution, and the range of disagreement was high (range of d values near 0.8). However, similar to mapping region results for PCA, the agreement between maps varies with regions (Fig. 7b). The comparisons of map S with the other three maps at 240 m resolution suggest that it has maximum agreement with the map K across all the mapping regions. The agreement of map S with B decreases in the eastern mapping regions (Fig. 7b).

#### Evaluative comparisons

Given that these maps are being compared post hoc, no experimental manipulations of method can be designed to unambiguously identify causes of change. However, by designing evaluative tests to compare specific maps against each other under specific hypotheses of change, we can paint a richer picture of possible sources of difference. Thus, we first describe the results of the metric comparisons here, and then follow with an assessment of how those comparisons should be interpreted on a pairwise basis to test potential methodological sources of error.

#### Cumulative biomass

The same cumulative biomass results conducted for the descriptive phase apply to the evaluative phase (Table 1; Fig. 3). Notably map S and map W agree well with FIA, and maps W and B have higher estimates. This contrast is likely related to the commonality of allometric equation (CRM approach) between the FIA and S and W maps.

#### Mapping regions scale biomass density

**Fig. 8:**
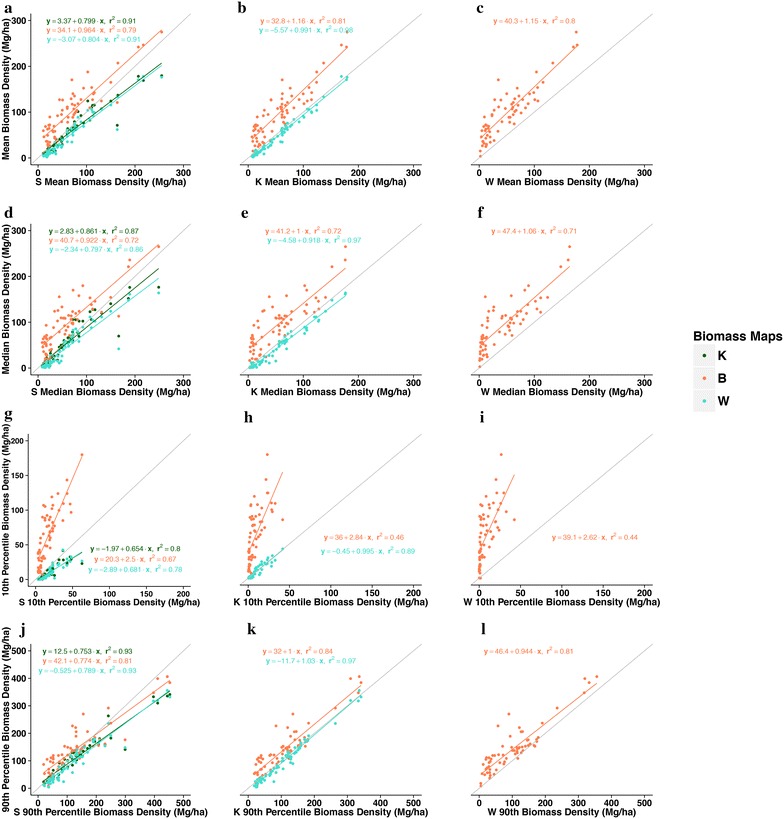
Pairwise comparison (least square regression) of mean and different percentiles of four AGB maps at mapping zone scale: mean (*first row*), median (*second row*), 10th percentiles (*third row*), and 90th percentile (*fourth row*) and each column in a row represents a particular AGB map as x-axis (**a**, **d**, **g**, **j**: S map as X axis; **b**, **e**, **h**, **k**: K map as X axis; **c**, **f**, **i**, **l**: W map as X axis)

Pairwise comparisons between the four maps at low, medium and high biomass density quantiles at mapping region scale parse out differences among maps more closely. In general, maps K and W track the 1:1 line at low, median, and high biomass, suggesting agreement across the biomass distribution and across all mapping regions (Fig. 8d, h, k). Map S agrees with Maps K and W at the median of the biomass distribution (Fig. 8d), but is generally higher than Maps K and W at both high and low ends of the range (Fig. 8g, j). Map B is higher than all other maps in nearly all situations, and particularly appears to have relatively higher biomass at the low end of the biomass range (Fig. 8g–i).

#### Difference maps and relation to ancillary data

**Fig. 9:**
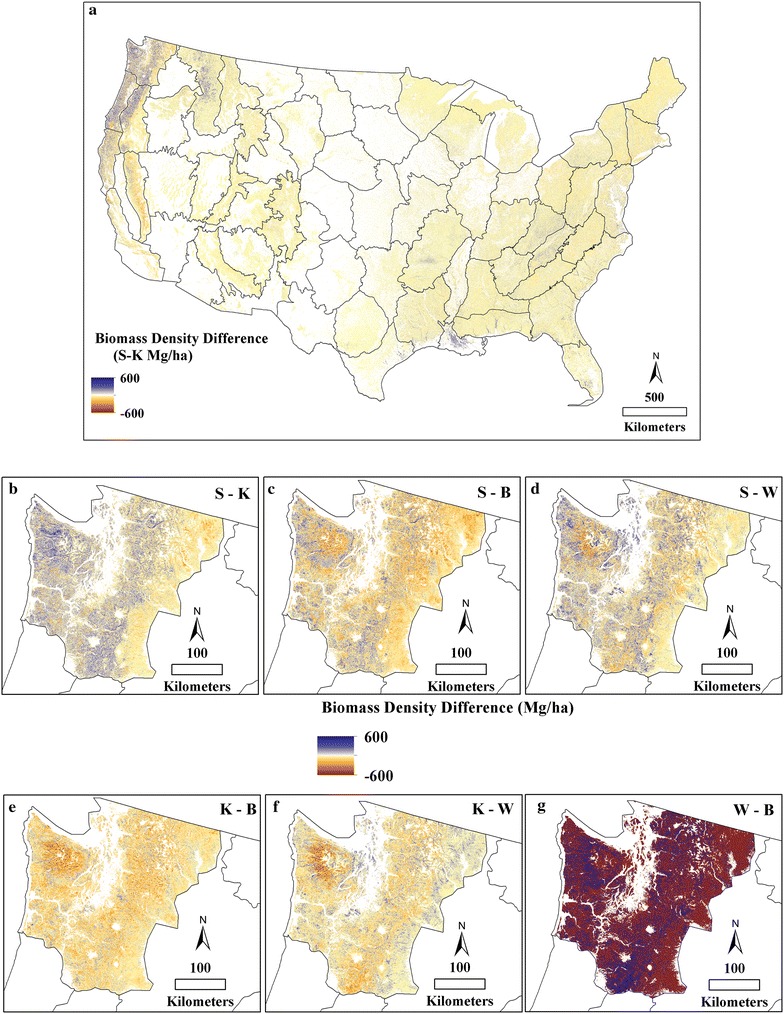
Pairwise differences between four AGB maps: **a** Difference between the map S and the map K for conterminous US, pairwise difference between AGB density maps in mapping zone 1: **b** S & K, **c** S & B, **d** S & W **e** K & B **f** K & W, and **g** W & B

**Fig. 10:**
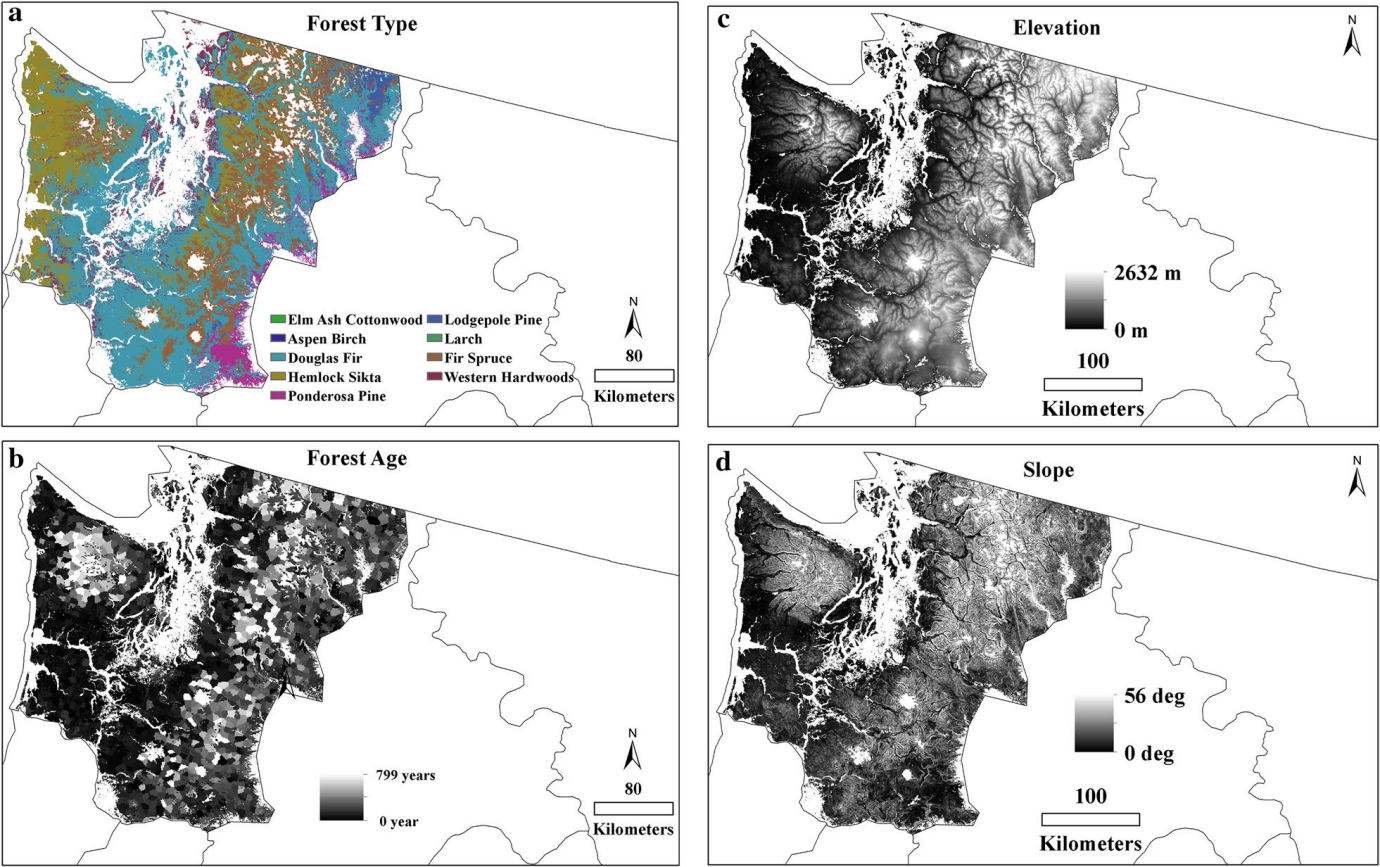
Forest and topographical characteristics for mapping zone 1: **a** forest type **b** forest age, **c** elevation, and **d** slope

**Fig. 11:**
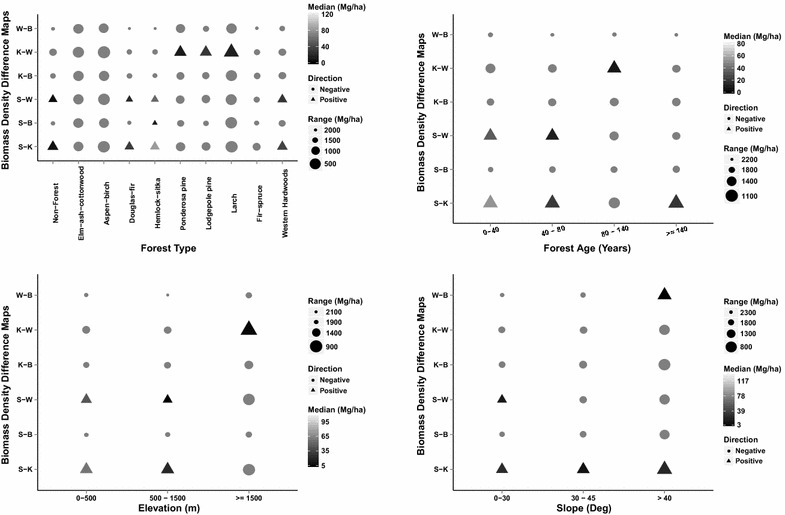
Relationship between pairwise AGB difference maps and various forest and topographic characteristics in mapping zone 1: **a** with forest type, **b** with forest age, **c** with elevation, and **d** with slope

We evaluated difference maps in relation to ancillary data for all 66 mapping regions. At the US scale, the difference between the map S and K suggests that the map S has higher biomass estimates compared to the map K at higher biomass regions, and lower biomass at lower biomass regions (Fig. 9a). Again, we use mapping region 1 to illustrate core principles and findings. In mapping region 1, Map S has higher biomass estimates than does Map K in the wetter, western part of the region, but that pattern is generally flipped when compared to Maps B or W (Fig. 9 b–d). Forest characteristics (Fig. 10a, b) and topography (Fig. 10c, d) were then related on a pixel by pixel level to the difference map, and difference median and range summarized by forest type (Fig. 11a), forest age (Fig. 11b), elevation (Fig. 11c), and slope (Fig. 11d). As before, Map B was an outlier in most comparisons in this mapping region. In most of the comparison with map B, the median biomass difference is farther from 0, the range of difference is larger with higher biomass estimates across the mapping region.

#### Testing sources of disagreement

Metrics of comparison described above are the core from which tests can be developed. Again, the goal of the evaluative comparisons is to use specific map-pair comparisons by metric to infer whether certain methodological choices are contributing to map disagreement.

#### Forest mask

If forest mask area were a fundamental reason for disagreement, we would expect maps with conservative forest area to show less biomass. But comparing total forest and total biomass (Section on cumulative biomass above, Table 2), there is little evidence that forest mask was an important factor driving the difference among maps. Indeed, the map with the highest biomass estimates (Map B) had the most conservative forest mask.

#### Spatial resolution

For tests of the impact of spatial resolution, we compared the high-resolution maps (S and K) with the coarser-resolution maps (W and B). If spatial resolution were an issue we would expect coarse resolution maps to show compressed data ranges at both high and low biomass values relative to the higher resolution maps. Map S appears to show this effect, as it shows greater range in both the 90th and 10th percentiles compared to the W map (Fig. 8j, g). However, map K did not show this effect in comparison to map W (Fig. 8h, k), suggesting that resolution alone does not explain the differences in the maps.

#### Source sensor data

If active sensors are able to map higher biomass without saturating, we would expect maps W and B to saturate at high biomass relative to both map S and K. Map S does appear to estimate higher biomass (90th percentile) than all other maps (Fig. 8j), but this effect does not occur with the other map created using an active sensor (Map K). However, this effect may be associated with the use of L-Band PALSAR in the map S. Map K uses C-band InSAR, which is less sensitive to the higher biomass compared to the higher wavelength [23]. Thus, our results are consistent with the notion that a specific type of active sensor may provide greater response to high biomass areas.

This pattern can be partially corroborated by focusing on the high-biomass mapping region 1 (Fig. 11a). There, map S shows higher biomass estimates compared to K (that uses C-band radar data for height measurement) for high biomass forests (Douglas fir or Hemlock/Sitka spruce; Fig. 11a). However, the contrast does not hold for high biomass age classes (>80 years old; Fig. 11b). Moreover, when compared to optical-based maps, the map K map does not consistently show higher biomass estimates in high biomass forests (Douglas fir or Hemlock/Sitka spruce; Fig. 11a) nor in high biomass age classes (>80 years old; Fig. 10b).

Moreover, we see no evidence for topographic impacts on radar-derived maps. If topographic conditions were an issue for side-looking radar, maps S and K might show dampened range in regions of higher topography. But instead, we find that the range of difference values between either the S or K maps and the other maps show no patterns with topographic condition (Fig. 11d). We acknowledge that these maps also began as higher resolution sources, and thus errors of topography may be offset by improvements from resolution.

#### Extrapolation technique

We see no evidence that extrapolation technique influenced differences among the maps. Again, we focus on the high and low end of the biomass range, and ask whether maps that use parametric or semi-parametric approaches (maps S and W) are compressed relative to counterparts that use nonparametric approachs (maps B and K). In this case, neither the S nor W map shows compressed ranges in the 10th or 90th percentiles of biomass distributions relative to the other maps (Fig. 8). This argues that extrapolation technique may not play a large role in determining differences among maps.

#### Nominal year of map

**Fig. 12:**
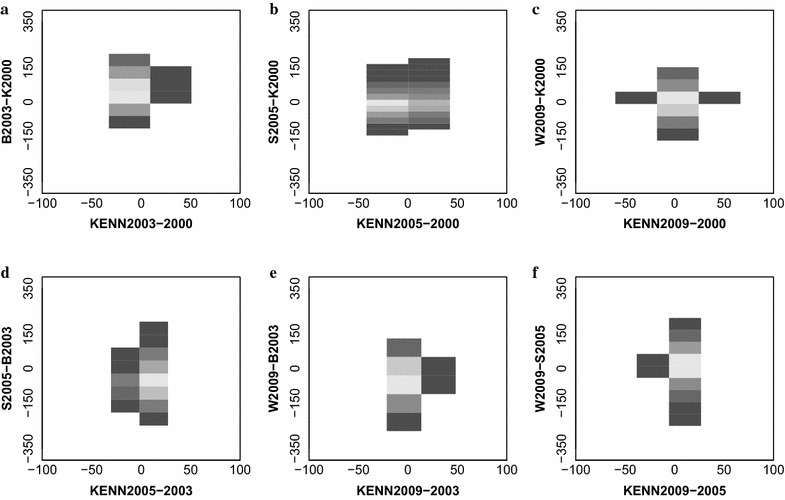
Relationship between change in AGB (Mg/ha) in the national scale maps and the regional maps for disturbances and regrowth pattern for the states of WA, OR and CA over the years: **a** between 2000 and 2003, **b** between 2000 and 2005, **c** between 2000 and 2009, **d** between 2003 and 2005, **e** between 2003 and 2009, **f** between 2005 and 2009

Because maps differed in nominal year represented, we would expect possible differences due to disturbance and growth of forests between map years. We tested against a regional scale map whose production focused on disturbance and growth, and that had consistent methods across time (Fig. 12). Although weak, evidence suggests that between 40 and 45 % of the total difference between national maps was consistent with growth and disturbance in the regional-scale map (Table 3).

**Table 3:** Total biomass estimates in million tonnes in three western states (OR, WA, and CA) over four years: Comparison between national map and regional map based estimates

States	2000 (Mt)	2003 (Mt)	2005 (Mt)	2009 (Mt)
WA	K: 1564.88	B: 2091.94	S: 1812.82	W: 1732.58
	Kenn: 1547.72	Kenn: 1537.92	Kenn: 1526.93	Kenn: 1509.06
OR	K: 1838.67	B: 2564.11	S: 2150.93	W: 1957.12
	Kenn: 1765.63	Kenn: 1734.37	Kenn: 1727.32	Kenn: 1708.72
CA	K: 2106.35	B: 2305.67	S: 1818.43	W: 1974.56
	Kenn: 1815.66	Kenn: 1808.30	Kenn: 1810.52	Kenn: 1798.77

#### Allometric equations

Allometric equation impacts may have played a role in some of the differences among maps. Median and mean biomass estimates from Map B (using regional scale equations) were consistently higher than all other maps (Fig. 8a–f), and Map W’s estimates (using CRM equations) were lower than Map K’s. However, Map S did not fit the pattern, showing higher median values than both Map K and W.

## Conclusions

There exist several AGB maps generated using different set of input data and methodology. Although these maps describe the same biophysical variable, they vary both in quantity and spatial pattern. We provide a systematic approach to describe and evaluate the differences between maps at multiple scales, while assessing the accuracy of individual mapping effort at an aggregated scale. This study emphasizes that comparison between maps need to be structured with specific hypothesis and tests. Moreover, we suggest that simple directives about which map to use are perhaps overly simplistic. Indeed, the answer for defining various tests depends on the need of the user and scale of comparison and therefore one needs to have set of methodology at different scale with different identified steps. The results from this systematic analysis on comparison of four national level AGB maps suggest that the absolute accuracy and spatial pattern of agreement vary with scale (both spatial resolution and spatial extent). Three (S, K and W) of the four maps largely agreed, and the two maps (K and W) generated with quite different methodological workflows agreed remarkably well. One map generated with high resolution, active sensor data appeared to capture a greater range of data. Finally, the map that did not agree at the pixel scale continued to disagree even with aggregation, indicating aggregation alone may not make maps similar.

When compared with FIA based AGB summed at national scale—which is also basis for national level carbon accounting—all four maps slightly overestimate biomass. However, the total summed AGB for map S is most similar to FIA based estimates, and would therefore argue that the map S could be chosen among the four maps for national level analysis. However, the map W is most similar to FIA based estimates when aggregated to the state scale. The comparison among biomass maps at pixel level (240 m resolution) using R-mode PCA suggests that spatial structure of agreement varies at national and regional scale (spatial extent). Map B disagrees most with respect to the other maps when analyzed at national scale, but it is not always true when analyzed at the mapping region scale. Thus, one needs to look at both regional and broad scale differences before making decision about using one map over others for carbon accounting. The spatial structure of the maps S and K have maximum agreement at national scale PCA analysis, thus they agree well with each other as well as FIA based estimates.

The spatial structure of agreement also varies with the spatial resolution. Except for map B, the agreement among other maps increases as spatial resolution coarsens. Maximum agreement occurs in the northwest (higher biomass) mapping region of the country. The changes in agreement with aggregation varied between maps, and were not same for the three maps (leaving map B aside).

The structure of the agreement and disagreement among the AGB maps achieved by testing the full suite of potential sources of differences provides evidence in support of causes of differences. For example, all the maps did best in the dominant forest type of a given region, but variability was found in non-dominant forest types. The comparison of maps with regional efforts provided information about how well these maps were able to capture regrowth and disturbance pattern. For example, the comparison of the difference between the maps K and S with regional map suggest that the locations where there is lower biomass in S compared to K is mainly due to disturbance. However, the difference between the two could not capture regrowth well. Thus, sequences of maps capture both actual change and the combined effects of each map’s error, and thus argues against using sequences of these maps for spatial monitoring of biomass over time.

Our strategy for testing the full suite of potential error sources shows how remarkably consistent some maps were. Notably, the W and K maps differed in nearly every methodological approach, yet agreed closely at the mapping region scale. The magnitude of W and K matches closely even though the spatial structure differs whereas the spatial structure of S and K agrees (simultaneous comparison using PCA) but magnitude differs (pairwise comparison) when compared at ecoregion scale.

While most of the maps generally agree at broad spatial scales, spatial patterns of disagreement at the local scale are notable. PCA analyses at both national and mapping region scale clearly show that the disagreement (PC2) has spatial pattern, and the improvement of agreement with spatial aggregation (Fig. 11) corroborates the notion that pixel-level estimates vary considerably among maps. Thus, a user at the regional scale would be advised to evaluate local scale variation among all maps before choosing one to get insight on the sources of differences (error or physical change in the landscape). With the understanding of differences, one can use ensemble approach to have an accurate map of aboveground biomass map.

Thus, this study provides guidance how to approach comparison of multiple maps systematically by designing specific steps for various hypothesis for describing and evaluating the spatial pattern of differences between maps.

## Abbreviations

NASA-CMS: National Aeronautics and System Administration Carbon Monitoring System
MSE: mean square error
RMSE: root mean square error
AGB: above ground biomass
FIA: Forest Inventory and Analysis
CRM: Component Ratio Method
PCA: Principal Components Analysis
ORNL: Oak Ridge National Laboratory
